# Emotional Distress, Cognitive Complaints, and Care Needs among Advanced Cancer Survivors Treated with Immune Checkpoint Blockade: A Mixed-Method Study

**DOI:** 10.3390/cancers16091638

**Published:** 2024-04-24

**Authors:** Nathalie Vanlaer, Iris Dirven, Bart Neyns, Anne Rogiers

**Affiliations:** 1 Department of Medical Oncology, Vrije Universiteit Brussel, Universitair Ziekenhuis Brussel, 1090 Brussels, Belgium; 2 Department of Psychiatry, Centre Hospitalier Universitaire Brugmann, 1020 Brussels, Belgium

**Keywords:** fear of cancer recurrence, cancer-related post-traumatic stress, cognitive function, care needs, psychological distress, survivorship issues, psychosocial functioning, cancer survivors, immune checkpoint blockade

## Abstract

**Simple Summary:**

Currently, there is very little data on survivorship-related issues after successful treatment by immune checkpoint blockade for an advanced cancer. The purpose of this study was to identify survivorship-related issues, with a focus on emotional distress, cognitive complaints, physical issues, impact on family dynamics, and care needs in survivors treated with immune checkpoint blockade therapy for an advanced cancer. We conducted semi-structured interviews and completed validated questionnaires with 70 survivors. We found that more than half of the cancer survivors had a clinical fear of cancer recurrence and that 18% had elevated cognitive complaints. We identified triggers related to severe emotional distress. Moreover, we found ongoing physical issues and unmet nutritional and emotional care needs. These results show that although patients are successfully treated, there are still ongoing psychosocial, physical, and cognitive issues and care needs that can be mitigated by routine screening and referrals to psychological services.

**Abstract:**

Background: There is a need for a better understanding of survivorship-related issues in advanced cancer survivors treated with immune checkpoint blockade (ICB). The purpose of this study was to identify survivorship-related issues, with a focus on psychological distress, cognitive complaints, physical sequelae, impact on family dynamics, and care needs in unresectable, advanced cancer survivors treated with ICB. Methods: Semi-structured interviews and patient-reported outcome measures (PROMs) were conducted in survivors followed up at the University Hospital Brussels. We performed content analysis on the semi-structured interviews and analyzed the PROMs descriptively. Results: 70 cancer survivors (71.4%) consented to participate between July 2022 and November 2023. Clinical fear of cancer recurrence (FCR) was present in 54.3% of the cancer survivors, and 18.6% had elevated cognitive complaints. We identified triggers related to clinically important psychological distress, such as immune-related adverse events, the progression/recurrence of disease, difficulties in adjusting to life after treatment, and co-existing life stressors, alongside persistent physical issues and unmet psychological and nutritional care needs. Conclusion: Our results indicate the existence of persistent psychological, physical, and cognitive issues, and support the need for routine screening for FCR. The identified triggers related to severe psychological distress can aid clinicians in timely referring the patient, thereby enhancing survivorship care.

## 1. Introduction

In the last 12 years, advances in the medical treatment of solid tumors and, in particular, the availability of effective immunotherapy in the form of immune checkpoint blockade (ICB) have enabled oncological patients with metastatic disease to enjoy disease-free survival. Historically, survivorship issues in patients with an advanced stage of cancer were not considered, given the patients’ poor prognosis and survival [1]. Since the registration of ipilimumab, an anti-cytotoxic T lymphocyte-associated antigen-4 (anti-CTLA-4) monoclonal antibody, in 2011 and the registration of monoclonal antibodies against programmed death-1 (PD-1) (e.g., pembrolizumab, nivolumab) and PD-L1 (e.g., atezolizumab) in 2014, survival rates have improved tremendously among patients with an advanced-stage cancer [2]. Recent registries show that more than half of the cancer survivors were diagnosed within the past 10 years, indicating the important progress that has been made in cancer treatment and prevention [3]. Given the increasing number of patients living beyond the acute treatment phase, there is a need to better understand survivorship issues of advanced- and/or metastatic cancer survivors [4]. A considerable number of patients on immune checkpoint blockade (ICB) therapy suffer from chronic immune-related adverse events (irAEs), defined as irAEs persisting for at least 12 weeks after stopping treatment [5]. A recent retrospective study in patients receiving anti-PD-1 ICB therapy in an adjuvant setting after resection of a high-risk melanoma found that 43% of the patients developed a chronic irAE, such as hypothyroiditis and arthralgia [5]. In metastatic melanoma treated with ICB, skin toxicity, arthralgia, and fatigue are among the most reported chronic toxicities [6]. In addition, long-term physical effects such as chronic pain and lymphedema are often reported across several cancer types and treatments [7].

Advanced cancer survivors, in general, have a higher level of uncertainty about their prognosis and progression-free-survival as compared to early-stage patients, and they tend to have a complex treatment history [8]. A considerable amount of advanced cancer patients are confronted during their disease trajectory with one or more recurrences. This implies important psychological challenges when dealing with a life-threatening diagnosis [1]. 

Knowledge of psychosocial outcomes, neurocognitive functioning, and health-related quality of life (HRQoL) remains scarce in this specific survivor population treated with ICB [9,10]. A survey study in metastatic melanoma patients treated with ICB and targeted therapy found that scan-related anxiety, fear of recurrence, and worries about dying were very frequent, ranging from mild to severe fear in 64–81% of patients treated with ICB [6]. In metastatic melanoma patients successfully treated with pembrolizumab, clinical levels of anxiety and/or depression were found in 64% of the survivors. A clinical interview among these survivors found that 56% worried daily about disease recurrence, and 48% suffered from cancer-related post-traumatic stress disorder [9]. In first-generation long-term metastatic melanoma survivors treated with ipilimumab, corresponding results were found in survivors suffering from an ongoing fear of cancer recurrence and post-traumatic stress as well as existential problems and survivor guilt [10]. Both studies were the first to study objective cognitive impairment, which was present in 32% of the survivors treated with pembrolizumab and 41% of the long-term survivors treated with ipilimumab [9,10,11]. 

Regarding health-related quality of life (HRQoL), conflicting results have been reported. In metastatic melanoma survivors, several studies showed long-term emotional and physical symptoms while maintaining their global QoL [10,12]. On the other hand, other studies demonstrated a lower global QoL compared with a healthy population [9,13]. A systematic review among unresectable, advanced melanoma survivors found inconsistent results in patients treated with ICB [14]. In non-small-cell lung carcinoma (NSCLC), these contradictions exist as well, where some report a similar HRQoL compared to the healthy population, while others report chronic physical toxicities and psychological concerns [15,16]. 

Currently, little is known about psychosocial functioning and HRQoL in advanced-stage cancer survivors treated with ICB. Earlier studies focused mostly on melanoma survivors treated with ICB and results of HRQoL in unresectable melanoma remains inconclusive. This study builds further on the earlier findings of our research group that melanoma survivors are susceptible to low emotional and cognitive functioning [9,10]. In this study, we expand this knowledge by including any type of advanced cancer treated with ICB and by performing a more comprehensive psychometric and qualitative evaluation of the survivorship-related issues. The objective of the study was to investigate survivorship-related issues, with a focus on psychological distress, cognitive complaints, physical sequelae, impact on family dynamics, and care needs in unresectable, advanced cancer survivors treated with ICB.

## 2. Materials and Methods

### 2.1. Study Participants and Procedures

This manuscript reports on a cross-sectional analysis of the baseline results of an ongoing multi-center cohort study (ClinicalTrials.gov: NCT05667857). The ongoing cohort study evaluates the following survivorship-related issues: neurocognitive functioning, psychosocial issues, and health-related quality of life in a longitudinal manner. This cohort study comprises three evaluations: one at baseline (T0), a second evaluation six months after baseline (T1), and one a year thereafter (T2). At baseline, a semi-structured interview, neuropsychological testing, and a series of patient-reported outcome measures (PROMs) are completed. During the follow-up assessments, the same PROMs and neuropsychological testing are performed as at baseline. In this manuscript, we report on the baseline results of the PROMs and the semi-structured interviews to evaluate psychological distress, cognitive complaints, and care needs. 

Patients diagnosed with unresectable stage III/IV cancer of any type, who initiated ICB at least one year prior to the time of inclusion, and who had a complete metabolic remission on whole-body 18F-fluorodeoxyglucose positron-emission tomography/computed tomography-([18F]-FDG PET/CT) were invited to participate. This study was approved by the institutional Committee of Medical Ethics of the Universitair Ziekenhuis Brussel (UZ Brussel) in 2022 (CME: 2022-043). All patients provided their written informed consent prior to study enrollment.

Patients were actively recruited during their oncological follow-up consultations in the UZ Brussel or were contacted by telephone. After agreeing to participate, the patients completed a baseline assessment (T0). Sociodemographic information was gathered through a general questionnaire. Information on medical history was retrieved through hospital patient files. 

#### 2.1.1. Semi-Structured Interviews

The semi-structured interviews were conducted by NV, a trained clinical psychologist, with the objective of examining how the patients’ psychosocial functioning related to the experience of their oncological trajectory. The semi-structured interview assessed how the patient experienced the moment of his/her diagnosis (questions 1–2), their certainty about nearing death (question 3), the wish to die during or after their illness (question 4), suicidal thoughts during or after their illness (question 5), the presence of concentration or memory complaints (question 6), changes in relationships with their partner or family (question 7), positive effects of being diagnosed with cancer (question 8), the most difficult moment during the disease (question 9), their current most burdensome symptoms (question 10), changes in nutrition and the need for nutrition guidance (question 11–12). During the interview, notes were taken as much as possible verbatim. Afterward, the regular steps of a content analysis were performed.

We opted to use both semi-structured interviews as well as patient-reported outcome measures (PROMs). The benefits of using semi-structured interviews are that they give in-depth information about the context of symptoms, such as triggers related to the psychological distress, as well as care needs, which are not detected by PROMs. Moreover, qualitative methods can provide additional information on items that might not be included in the questionnaires. In contrast to auto-evaluations by patients using the PROMs, semi-structured interviews are clinician-led and are dependent on the interview style. In this study, all semi-structured interviews were performed by the same person, a trained clinical psychologist, to counter inter-rater variability and to warrant the quality of the interviews. The PROMs provide the possibility of measuring the severity of the psychosocial variables and correlations between the variables, and they enable comparisons with other groups and other studies using the same PROM. 

#### 2.1.2. Patient-Reported Outcome Measures

**Fear of cancer recurrence.** We measured the severity of the fear of cancer recurrence (FCR) through the Fear of Cancer Recurrence Inventory—Short Form (FCRI-SF). This validated scale consists of nine items with a range of 0–36, with higher scores indicating greater severity [17,18]. We used the recommended cutoff of ≥13, which has good sensitivity (88%) and specificity (75%), to detect clinical FCR [19,20]. We also reported two other cutoff points provided by the original authors: ≥ 16, which has higher specificity, and ≥22, which evaluates pathological FCR [18]. The FCRI-SF has a strong correlation with the total FCRI score of the full questionnaire (r = 0.84), has a high internal consistency (α = 0.89), and has adequate stability over time (1 month = 0.80). 

**Cancer-related post-traumatic stress.** We measured post-traumatic stress through the post-traumatic stress disorder (PTSD) checklist for the DSM-5 (PCL-5) [21,22]. This is a 20-item questionnaire with a 5-point Likert scale, with a range of 0–80. It is a screening instrument for PTSD according to the DSM-5 criteria. A cutoff score of 33 has been proposed for the updated PCL-5 checklist, which has a sensitivity of 0.93 and specificity of 0.72 [23]. To evaluate if the post-traumatic stress was cancer-related, we asked patients to interpret the stressful experience that the questionnaire refers to as a stressful experience during the oncological trajectory. In that way, we avoided patients performing the self-evaluation based on an experience that was not related to cancer.

**Subjective cognitive complaints.** The Cognitive Failures Questionnaire (CFQ) is a 25-item validated questionnaire on a 5-point Likert scale that measures lapses in cognition in everyday tasks [24]. The values of the CFQ range between 0–100, with higher scores indicating more subjective cognitive complaints. An optimal cutoff point of ≥44 or ≥55 is identified to assess, respectively, moderate or severe subjective cognitive complaints [25]. It has a good internal consistency, with α = 0.88, and is validated both in Dutch and French [25,26].

### 2.2. Statistical Analysis

There were no missing values. All PROMs were verified for completeness during the evaluations. Statistical analysis was performed using SPSS running on version 29.0.0.0 (IBM, Armonk, NY, USA). All PROMs were analyzed descriptively. As mentioned above, we compared the PROMs with their clinical cut-off scores. A Pearson correlation analysis was conducted between all PROMs and age, as well as two-sided independent t-tests between gender and FCR, and gender and CFQ (significance level at 0.05). Due to a non-normal distribution of the PTSD-checklist for the DSM-5 outcomes, we analyzed the gender differences in PTSD symptoms using the Mann–Whitney U test. To analyze the qualitative data of the semi-structured interview, we performed an inductive content analysis according to Elo and Kyngas [27]. The qualitative data yield a supporting explanation to the outcomes of the PROMs. 

## 3. Results

### 3.1. Study Population

Of the 98 patients considered, 70 patients (71.4%) consented to participate between July 2022 and November 2023 (see Supplementary Figure S1). Forty-two patients were males (60.0%). The median age was 65.0 years (range 34–92). The majority (n = 57; 81.4%) were treated for melanoma. Sixty-three patients (90.0%) had a history of a stage IV disease, among whom 13 (18.6%) had a history of brain metastasis. Almost all patients were off treatment (n = 67; 95.7%). Forty patients (57.1%) were long-term survivors with over 5 years of survivorship, counted from the start of ICB administration. The median time since complete remission was 3.3 years (range 0.0–13.3). Forty patients (57.1%) received at least two lines of therapy. Patient characteristics are summarized in Table 1.

### 3.2. Semi-Structured Interviews

During the semi-structured interviews, contextual factors were discussed that could give rise to the psychosocial issues. See Table 2 for a summarized scheme of the content analysis.

#### 3.2.1. Cancer as a Traumatic Experience

Almost half of the cancer survivors (n = 31; 44.3%) reported that they were certain that they would die at a certain point during their oncological trajectory. Triggers that gave rise to this certainty of dying were the diagnosis of metastasis (n = 10), the initial progression or recurrence of disease (n = 8), the treating oncologist communicating the exhaustion of approved treatment options (n = 6), grade 2 and 3 irAEs (n = 5) (G2 pneumonitis in one NSCLC patient; G2 hypophysitis with severe fatigue and anorexia in two patients (G2 fatigue in one patient, G3 in the other); G3 colitis in two patients), and pain and physical complaints of bone metastasis (n = 1).

One patient illustrated the diagnosis of metastasis as follows: “*When I received the diagnosis of brain metastasis, being alive hurt. I had two young children at the time. I received brain irradiation, which wasn’t successful. My initial oncologist could not help me further. I took a symbolic farewell.*” (patient 35)

In patients who did not feel certain about nearing death, we identified having confidence in the treatment and in the oncologist (n = 10), undergoing the treatment in a detached way (n = 4), and mentally focused on surviving (n = 3) as coping strategies. Other patients reported having thought about the end of life and euthanasia in preparation for disease progression (n = 8).

A patient explained: “*In my head I was 100% sure of surviving because there still was a probability of treatment. I was there to survive.*” (patient 12)

Another patient reported: “*I was not certain that I would die, but the idea of the last days, end- of life, came to my mind. I thought about euthanasia if the disease progressed. I didn’t want to end up like [family member] who died of cancer and suffered.*” (patient 70)

Eleven patients (15.7%) reported a desire to die at a certain point during or after the oncological treatment. The wish to die was related to grade 2 and 3 irAEs (n = 3) (G2 arthralgia, headaches and xerophthalmia related to previous vemurafenib treatment; G2 hypophysitis with severe fatigue and anorexia related to ipilimumab + nivolumab and pembrolizumab), progression/recurrence (n = 3), adjusting to life after cancer treatment after achieving remission (n = 2), and co-existing life stressors such as psychosocial and relational issues (n = 2) and pain and severe physical disability due to bone metastasis (n = 1). Three out of the eleven patients that reported having a wish to die during or after the oncological treatment, also had an indication for cancer-related PTSD.

Five patients (7.1%) reported suicidal ideations during or after the oncological treatment that was related to adjusting to “normal” life when being in remission (n = 2), co-existing life stressors such as relational and financial issues (n = 2), and recurrence of the disease (n = 1). For three of the five patients, suicidal ideation was characterized by momentary passive suicidal thoughts. Two patients had active suicidal thoughts during the treatment, including one patient who made a suicide attempt during the treatment. One patient reported having recurrent periods of passive suicidal thoughts at the present time. 

A patient who experienced suicidal ideation due to the difficulties in adjusting to “normal” life explained: “*One year after being in remission, I didn’t accomplish anything except surviving. The universe intended me to die, but I didn’t. I didn’t find my place back in society. During the treatment I had a goal to conquer. After the treatment, this goal was gone.*” (patient 31).

Forty patients (57.1%) experienced positive effects of living with the disease history of cancer. Fourteen patients (20.0%) reported having a mindful mindset in which they enjoyed the here-and-now and the small things in life more, thirteen patients (18.6%) experienced personal growth, through either getting to know themselves better, feeling more empathic toward others, or being more attentive to self-care. Eleven patients (15.7%) indicated a changed perspective toward their values of life. In doing so, patients noted putting things more into perspective. Four patients (5.7%) reported spending more quality time with their family and/or spouse. Twenty-four patients out of the 40 patients (60.0%) that experienced positive effects also had a clinical fear of cancer recurrence, indicating that patients can experience positive effects from the disease while enduring psychological distress.

One patient described these positive effects as: “*You learn about yourself and about each other. For example, how to cope with anxiety. You also appreciate the little things in life more and you live more in the moment.*” (patient 16)

Another patient described: “*I relativize more. I am more conscious of the value of life since the treatment and I try to not follow the “rat race” of society anymore.*” (patient 20)

#### 3.2.2. Changes in the Relationship with Spouse or Family

The oncological trajectory resulted in changes in the relationship with their partner or family members in 38 patients (54.3%). Nineteen patients (27.1%) reported a positive change in the relationship, manifested in becoming closer to their partner or family members (n = 13; 18.6%) and/or the partner or family members becoming more caring with the patient (n = 7; 10.0%). Eight patients (11.4%) reported both a negative and positive impact on the relationship, based on becoming closer to a certain family member while becoming more distant to another family member from whom the patient received little support or understanding (n = 5; 7.1%) or becoming closer to the family members while also putting more burden on the family members (n = 3; 4.3%). Moreover, eleven patients (15.7%) reported a negative impact of the disease on the relationship with their spouse and/or family. Reasons for a negative impact were a continued emotional and physical burden of the patient on the family member and/or spouse (e.g., spouse has to take over household tasks because of chronic fatigue), the patient becoming more easily irritable and less tolerant since the disease, differences in values of life, and not having received support during the treatment. 

“*We became closer as family, but it was also a stressful and traumatic experience for the whole family. It was also a burden for my partner, and still is due to my fatigue.*” (patient 4)

#### 3.2.3. Most Burdensome Symptoms

During the semi-structured interviews, chronic fatigue (n = 30, 43.9%) followed by lymphedema (n = 7, 10.0%) and cancer-related mobility problems (n = 7, 10.0%), psychological distress (n = 7, 10.0%), arthralgia (n = 6, 8.6%), cognitive complaints (n = 4, 5.7%), and dyspnea (n = 4, 5.7%) were reported as the current most invalidating symptoms. Ten patients (14.3%) did not experience any complaints. The above-mentioned mobility problems were related to a history of bone metastasis or the cancer treatment (radiotherapy, surgery, post-limb perfusion, or presumed treatment-related spinal disc herniation).

Regarding the fatigue, one patient illustrated that: “*I have to constantly take this [fatigue] into account and I need to plan everything beforehand. For example, not being able to dine with friends in the evening because of the fatigue.*” (patient 4)

#### 3.2.4. Cognitive Complaints

During the interview, thirty patients (42.9%) reported having memory and/or concentration problems that impacted on their daily life activities. Among those, most patients reported both concentration and memory complaints (n = 13; 18.6%), while others reported solely concentration problems (n = 12; 17.1%) or memory problems (n = 5; 7.1%). Some patients connected their cognitive problems to fatigue (n = 7; 10.0%). Among the activities that were impaired due to cognitive problems, most patients reported difficulties with reading a book or newspaper (n = 17; 24.3%), doing household tasks (n = 12; 17.1%), doing job-related tasks in the workplace (n = 10; 14.3%), doing hobbies (n = 7; 10.0%), following a TV series or movie (n = 6; 8.6%), and driving (n = 3; 4.3%). An overview of the cognition-related diminished functioning in daily life activities can be found in Table 3.

#### 3.2.5. Care Needs

Six patients (8.6%) spontaneously accentuated the importance of psychological care both during and after the oncological treatment. Two of these patients also expressed the importance of close psychological follow-up when patients are in remission and the frequency of the oncological consultations reduce. 

One patient explained that: “*I had only one consult with a psychologist. The doctors can push more to get psychological support. Also, the moment when you are “set free” from the hospital when the treatment stops was difficult to me.*” (patient 11)

We asked the patients if they currently commit more to a healthy nutritional pattern than before the cancer. Twenty-six (37.1%) patients reported that they currently have a healthier diet than before, and 11 patients (15.7%) reported already eating healthily beforehand. Eighteen patients (25.7%) desired a nutritional follow-up or general advice. Most of the patients (n = 13; 18.6%) preferred receiving general information about a healthy nutritional pattern, such as examples of healthy and affordable nutrition. Three patients (4.3%) indicated a desire to receive nutritional advice on how to lose weight while also taking into account their specific medication (such as hydrocortisone). One patient (1.4%) desired nutritional advice to influence his/her chronic fatigue. Two patients (2.9%) started a nutritional intervention with the onco-nutritionist of the hospital after the baseline evaluation. All other patients preferred having a group-based general nutrition information session rather than an individual follow-up.

### 3.3. Patient-Reported Outcome Measures

#### 3.3.1. Fear of Cancer Recurrence and Cancer-Related Post-Traumatic Stress

Clinical fear of cancer recurrence was present in about half of the patients (n = 38; 54.3%). Moreover, 15 patients (21.4%) had a pathological fear of cancer recurrence, indicating the need for a specialized intervention for fear of cancer recurrence. The PTSD checklist for the DSM-5 indicated cancer-related PTSD in five patients (7.2%). In Table 4, the outcomes of the PROMs are summarized.

#### 3.3.2. Subjective Cognitive Complaints

According to the CFQ, 13 patients (18.6%) had elevated cognitive complaints, out of whom six patients (8.6%) had severe cognitive complaints. During the semi-structured interview, we examined in what manner the cognitive complaints manifested themselves in daily life, which is presented in Table 3.

#### 3.3.3. Correlation Analysis

**Figure 1:**
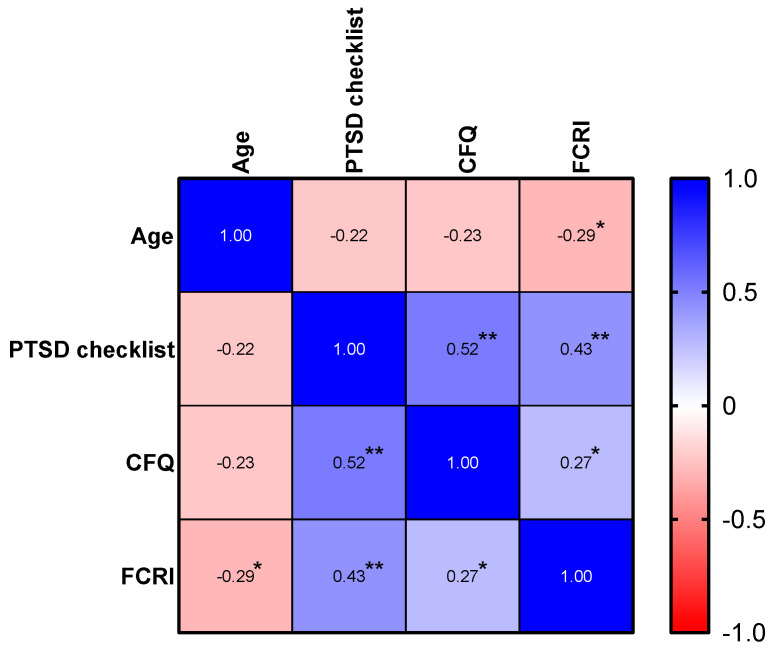
Heatmap of the correlation matrix between PROMs and age. Brighter colors represent a stronger correlation. The numbers in the boxes correspond to the Pearson correlation coefficient. One asterisk above the correlation coefficient represents a *p*-value lower than 0.05, two asterisks above the correlation coefficient represents a *p*-value lower than 0.001. A blue color represents a positive correlation, while a red color represents a negative correlation.

**Figure 2:**
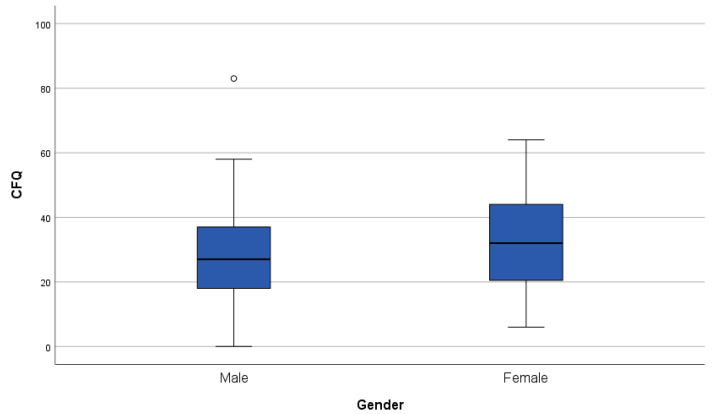
Boxplot of the Cognitive Failures Questionnaire (CFQ) outcomes grouped per gender. A higher score on the CFQ represents more cognitive complaints. The line in bold in the boxplot is the median. The dot in the figure corresponds to an outlier.

**Figure 3:**
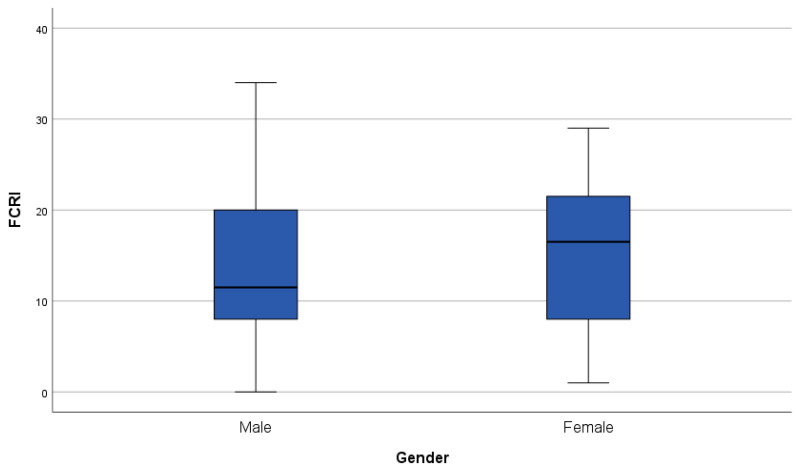
Boxplot of the Fear of Cancer Recurrence Inventory—Short Form (FCRI-SF) outcomes grouped per gender. A higher score on the FCRI-SF represents a higher severity of fear of cancer recurrence. The line in bold in the boxplot is the median.

**Figure 4:**
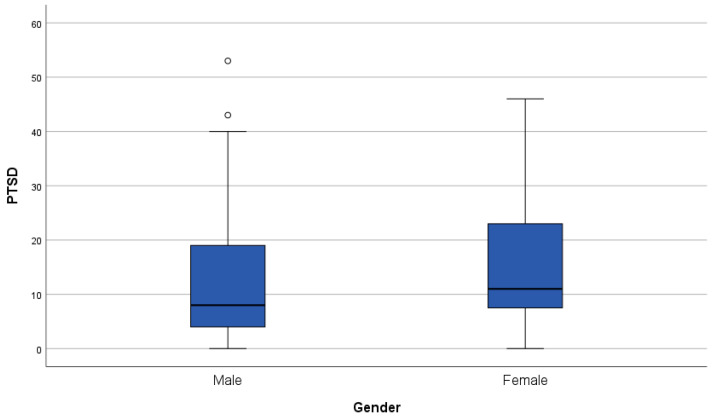
Boxplot of the Post-Traumatic Stress Disorder (PTSD) DSM-5 checklist outcomes grouped per gender. A higher score on the PTSD checklist represents a higher severity of PTSD symptoms. The line in bold in the boxplot is the median. The dots in the figure correspond to outliers.

All PROMs are significantly and positively correlated with each other (all *p*-values <0.05). Regarding demographical variables, age correlated significantly and negatively with FCR (*p* = 0.015, r = −0.288) but not with cognitive complaints or PTSD symptoms (see Figure 1). We observed no significant gender differences in any of the PROMs. Boxplots of the distribution of the PROMs per gender group can be found in Figure 2, Figure 3 and Figure 4. 

## 4. Discussion

In this cross-sectional analysis of the baseline results of an ongoing cohort study investigating survivorship-related issues in advanced cancer patients successfully treated with immune checkpoint blockade, we identified a high burden regarding the fear of cancer recurrence in a majority of the patients (54.3%) as well as cognitive complaints (18.6%). We identified unmet needs for nutritional and psychological guidance during and after the oncological treatment. A considerable number of patients reported a wish to die (n = 11; 15.7%) and had suicidal ideation (n = 5; 5.7%) during or after the treatment. In an earlier study of our research group, 28% of the metastatic melanoma survivors treated with pembrolizumab developed suicidal ideations [9]. We identified a number of triggers related to suicidal ideation and having a wish to die, such as disease progression/recurrence, difficulties in adjusting to a normal life after achieving remission, co-existence of life stressors during the disease trajectory, severe physical disability with pain, and grade 2 and 3 irAEs (e.g., hypophysitis with severe fatigue and anorexia). According to the biopsychosocial diathesis–stress model described by Schotte et al. [28], psychological dysfunction can be understood as a concept of vulnerability, such as biological, psychological, and societal risk factors and protective factors. Life stressors interact with the risk factors, which can lead to chronic distress. This affects the protective factors, leading to psychological dysfunction. In an oncological context, the burden of cancer-related stressors and the co-existence of life stressors (e.g., relational problems and financial issues), in combination with an already existent biogenetic vulnerability, may affect the protective factors (e.g., coping style, resilience, and the perception of receiving social support) and, subsequently, lead to psychological dysfunction and even suicidal ideation.

We measured cancer-related post-traumatic stress disorder quantitatively, with five survivors (7.1%) indicating post-traumatic stress disorder. This percentage of post-traumatic stress is particularly low compared with the percentage found in our two pilot studies, in which 35% and 48% of the patients were classified with cancer-related PTSD through a psychiatric interview [9,10]. We hypothesize that the increased number found in these pilot studies is related to the fact that these pilot studies investigated first-generation metastatic cancer survivors. These first-generation survivors had a very poor prognosis, as at the time of the study, it had not yet been unequivocally demonstrated that they could survive a metastatic disease, since there was no data on long-term survival. Therefore, it can be hypothesized that the announcement of the diagnosis was more traumatic at that time. Another possible explanation could be that the PTSD checklist for the DSM-5 does not adequately capture post-traumatic stress disorder and that there is a need for a more sensitive cancer-specific questionnaire or the use of in-depth psychiatric interviews to detect cancer-related PTSD.

The high percentage of survivors with clinical fear of cancer recurrence is in accordance with the two pilot studies of our research group that assessed FCR qualitatively. In these pilot studies, all survivors reported fear of cancer recurrence, and 47–56% experienced daily worrying about the disease [9,10]. In the current study, we assessed the fear of cancer recurrence quantitatively since there is limited data on patient-reported FCR outcomes in advanced cancer survivors successfully treated with ICB [29]. One interventional study on FCR in metastatic melanoma patients included the FCRI-SF in the screening phase. They reported a prevalence of 64% of clinical FCR (≥13) and 20% of pathological FCR (≥22), which is in line with the 54.3% and 21.4%, respectively, in our study [29]. These results underscore the high prevalence of FCR in advanced-stage cancer survivors and, subsequently, the need for structural screening throughout the survivorship trajectory, which accords with the guidelines of the American Society of Clinical Oncology on anxiety and depression [30]. In this context, a recent implementation study on a FCR intervention indicated good efficacy in reducing FCR as well as a high adherence and acceptability [31].

In addition, we found a significant positive correlation between FCR, cognitive complaints, and post-traumatic stress symptoms, as well as a significant negative correlation between age and FCR, indicating that younger patients were more inclined to suffer from FCR. No significant gender differences between the PROMs were found. This is consistent with a recent meta-analysis on correlates of FCR [32].

More than half of the survivors (54.3%) reported experiencing changes in family relations, with either a positive (18.6%), negative (11.4%), or both a positive and negative change (11.4%) in the relationship with different family members. When both positive and negative changes were reported by a patient, these changes in family relations depended on either the level of support received or the ambivalent feeling of being of burden to a family member while also feeling closer to them. The important social impact of the disease was also found in an earlier study on advanced melanoma survivors treated with ICB [9].

Moreover, the majority (57.1%) reported a positive impact by their disease history, reflected in personal growth, a changed perspective toward life values, or more quality time with family members. Importantly, this positive impact can co-exist with the above-mentioned issues, e.g., 60% of the patients that reported a positive impact also had clinical fear of cancer recurrence. The notion of personal growth and changed values of life can be understood in the context of post-traumatic growth, which is defined as the psychological changes that arise when confronted with a major life crisis or traumatic event [33]. These results correspond to a recent qualitative study in long-term responders treated with ICB or targeted therapy, in which they reported coping strategies such as reclaiming a sense of control, altering their perspective, or reshaping their lives toward their values [34].

Regarding physical complaints, most survivors reported chronic fatigue (n = 30; 43.9%), followed by lymphedema (n = 7; 10.0%) and cancer-related mobility problems (n = 7; 10.0%), psychological distress (n = 7; 10.0%), arthralgia (n = 6; 8.6%), cognitive complaints (n = 4; 5.7%), and dyspnea (n = 4; 5.7%) as the most invalidating symptoms. Ten patients (14.3%) did not have any physical complaints. Earlier research in advanced melanoma patients found that skin toxicity, arthralgia, and fatigue were among the most reported chronic toxicities related to ICB [6]. Our study adds to this information by highlighting the most invalidating symptoms as reported by the patient. The emphasis by patients on fatigue, lymphedema, mobility problems, and psychological distress as the most chronic invalidating symptoms can be of clinical relevance during follow-up consultations. During a medical follow-up visit, the most important aspect is generally the discussion of the imaging results. When a scan shows a persistent remission, the patient might find it no longer appropriate to discuss his/her emotional and/or physical discomfort.

According to the CFQ, we found that 18.6% of the cancer survivors have cognitive complaints. This stands in contrast to the semi-structured interview, where 42.9% reported having cognitive complaints. The higher prevalence in the interview can be explained by the broad interpretation of cognitive complaints. As such, survivors indicated having difficulties in reading, doing household tasks, doing job-related tasks in the workplace, doing their hobbies, following a TV series, and driving. Preliminary studies indicate that cancer-related cognitive complaints might not be limited to chemotherapy but can also be associated with ICB [11,35].

The limitations of the study consist of the heterogeneity in relation to years of survivorship and the treatment lines. We explicitly chose a broad scope and opened up the study to any type of cancer treated with ICB. A cohort analysis was not possible given the high number of melanoma survivors compared with other cancer types. These numbers reflect the current treatment landscape and the higher number of survivors of melanoma compared with other advanced-stage cancers. Follow-up assessments of this study are still ongoing and will be conducted at 6 months and 18 months from baseline.

The strengths of the study include the mixed-method design in evaluating survivorship, the broad evaluation of several aspects of survivorship issues, and the relatively high sample size in the context of a mixed-method design using semi-structured interviews.

Further studies should assess the impact of surviving an advanced-stage cancer among family members of the patients, as well as adequate screening tools for cancer-related post-traumatic stress disorder, with clinical cut-off points. 

Given the high number of cancer survivors with emotional distress, more awareness should be created in oncological centers. Specific cancer survivor programs based on these findings can aid in identifying and referring patients with important emotional issues such as having a wish to die, PTSD symptoms, or suicidal ideation.

## 5. Conclusions

A high proportion of unresectable, advanced cancer survivors treated with ICB have clinical FCR and physical and cognitive issues that impact on their daily life situations, as well as psychological and nutritional care needs. We identified triggers related to clinically important psychological distress, such as immune-related adverse events, progression/recurrence of disease, pain and physical complaints due to metastasis, difficulties in adjusting to life after treatment, and co-existing life stressors, which can help clinicians in the detection and referral of severe psychological distress. Routinely screening for FCR, timely referral to psychological services, and the implementation of a personalized survivorship care plan might mitigate these issues. Further research should focus on adequate screening tools and the impact on the wellbeing of relatives of the patient.

## Figures and Tables

**Table 1:** Descriptive characteristics of the study population.

Variables	n = 70 (100%)
Sex	
Male	42 (60.0%)
Median age in years (range)	65.0 (34–92)
Education	
Primary school	3 (4.3%)
Lower secondary school	8 (11.4%)
Higher secondary school	21 (30.0%)
Vocational education	7 (10.0%)
Higher education	31 (44.3%)
Social status	
Professionally active	29 (41.4%)
Sick leave	6 (8.6%)
Retired	35 (50.0%)
Civil status	
Married or cohabiting with partner	59 (84.3%)
Divorced, single	4 (5.7%)
Single, never married	5 (7.1%)
Widowed	2 (2.9%)
Children	
Young children at diagnosis (<18 y.o.)	18 (25.7%)
Adult children at diagnosis (>18 y.o.)	41 (58.6%)
No children	11 (15.7%)
Psychiatric history	
Depression	3 (4.3%)
Alcohol dependency	1 (1.4%)
No psychiatric history	66 (94.3%)
Psychotropic treatment	
Benzodiazepine	5 (7.1%)
Antidepressant	7 (10.6%)
Hypnotic Z-drug	1 (1.5%)
No psychotropic treatment	57 (81.4%)
Treatment and disease history	
ICB treatment	
Pembrolizumab	36 (51.4%)
Ipilimumab + nivolumab (incl. nivolumab maintenance)	13 (18.6%)
Nivolumab	9 (12.9%)
Ipilimumab	5 (7.1%)
Ipilimumab + dendritic cell therapy	5 (7.1%)
Atezolizumab	1 (1.4%)
Avelumab	1 (1.4%)
Line of therapy	
1st line	30 (42.9%)
2nd line	23 (32.9%)
3rd line	14 (20.0%)
>3rd line	3 (4.3%)
Previous systemic treatment before ICB	
Other ICB	18 (25.7%)
BRAF/MEK inhibitor	17 (24.3%)
Chemotherapy	14 (20.0%)
Study drug (dendritic cell therapy or IFN)	9 (12.9%)
Cancer type	
Melanoma	57 (81.4%)
Non-small-cell lung carcinoma	7 (10.0%)
Colorectal carcinoma	2 (2.9%)
Renal-cell carcinoma	2 (2.9%)
Transitional-cell carcinoma	1 (1.4%)
Merkel-cell carcinoma	1 (1.4%)
AJCC 8th edition cancer stage	
Melanoma	
IIIC	4 (5.7%)
IV-M1a	7 (10.0%)
IV-M1b	14 (2.0%)
IV-M1c	21 (30.0%)
IV-M1d	11 (15.7%)
Non-small-cell lung carcinoma	
IIIA	1 (1.4%)
IIIB	1 (1.4%)
IVA	3 (4.3%)
IVB	3 (4.3%)
Colorectal carcinoma	
IV-M1c dMMR/MSI-high	2 (2.9%)
Transitional-cell carcinoma	
IV-M1b	1 (1.4%)
Renal-cell carcinoma	
IV	2 (2.9%)
Merkel-cell carcinoma	
IIIB	1 (1.4%)
Brain metastasis	13 (18.6%)
Localized treatment for brain metastasis	
Stereotactical radiotherapy	8 (11.4%)
Surgery	2 (2.9%)
Surgery + post-surgical stereotactical radiotherapy	1 (1.4%)
No localized treatment	2 (2.9%)
Reason for stopping ICB treatment	
irAE	27 (38.6%)
Elective	40 (57.1%)
ICB ongoing	3 (4.3%)
Years of survivorship since time of first ICB administration	
≥5 years	40 (57.1%)
3–4 years	18 (25.7%)
1–2 years	12 (17.1%)
Median years since starting ICB administration (range)	5.1 (1.0–14.4)
Median years since the last ICB administration (range)	3.3 (0.0–11.3)
Median years since complete metabolic remission (range)	3.3 (0.0–13.3)
Median duration of ICB treatment in months (range)	13.7 (0.6–68.7)

**Table 2:** Results of the content analysis.

Theme	Category	Subcategory
Cancer as a traumatic experience	Contextual triggers of the certainty of dying	Diagnosis of metastasis Initial progression or recurrence of disease Oncologist communicating exhaustion of treatment options Grade 2 and 3 irAEs Pain and physical complaints due to bone metastasis
	Coping strategies	Having confidence in the treatment and oncologist Undergoing the treatment in a detached way Mentally focusing on surviving
	Contextual triggers of the wish to die	Grade 2 and 3 irAEs Progression/recurrence of disease Difficulties in adjusting to “normal” life after achieving remission Co-existing life stressors Pain and physical complaints due to bone metastasis
	Contextual triggers of suicidal ideation	Difficulties in adjusting to “normal” life after achieving remission Co-existing life stressors Recurrence of disease
	Positive impact of cancer diagnosis	Mindful mindset Personal growth Changed perspective toward values of life More quality time with family and spouse
Changes in relationship with spouse or family	Positive change	Becoming closer in relationship with partner or family Partner or family becoming more caring with patient
	Both negative and positive impact	Not feeling emotional supported or not feeling understood Becoming closer while putting more burden on family
	Negative impact	Continued emotional and physical burden of the patient on the family Patient becoming more easily irritable and less tolerant Differences in values of life Not feeling emotional supported during the treatment
Most burdensome symptoms	Physical symptoms	Chronic fatigue Lymphedema Cancer-related mobility problems Arthralgia Dyspnea
	Emotional and cognitive complaints	Psychological distress Cognitive complaints
Cognitive complaints	Diminished daily life activities	Difficulties with reading a book or newspaper Doing household tasks Doing job-related tasks in the workplace Doing hobbies Following a TV series or movie Driving
Care needs	Psychological care	Continued psychological care during and after treatment
	Nutrition	General information on healthy nutrition Nutritional advice on losing weight, taking into account specific medication Nutritional advice to influence chronic fatigue

**Table 3:** Overview of diminished functioning in daily life activities due to cognitive problems.

Diminished Daily Life Activities	Number of Patients Reporting Difficulties (n, %)	Examples from Patients
Household tasks	12 (17.1%)	- Multitasking, but not finishing any of the household tasks in the end (patient 2) - Forgetting groceries (patient 44)
Following a TV series or movie	6 (8.6%)	- Falling asleep when watching a series (patient 4) - Not being able to follow the series due to distraction (patient 59)
Professional context	10 (14.3%)	- Difficulties with adapting to changes in the work environment (patient 28) - Wanting to complete multiple things at the same time without success, tendency to chase myself at work (patient 28) - Everything takes more time, I’m easily distracted and I remember less well (patient 48) - Not starting to work as an independent, which was my dream job, because of the fear of forgetting appointments and not being able to handle the pressure (patient 31)
Reading a book or newspaper	17 (24.3%)	- Having to re-read pages (patient 37) - Difficulties to remember what I read and therefore not enjoying it (patient 39)
Doing hobbies	7 (10.0%)	- Problems initiating the activity because of less motivation and enjoying less (patient 37)
Driving	3 (4.3%)	- Not daring to drive a route that I don’t know by heart (patient 57) - Not driving during the night because of fatigue (patient 4)

**Table 4:** Outcomes of the patient-reported outcome measures.

	Cancer Survivors n = 70
**Post-traumatic stress (PTSD checklist for DSM-5)**	
Mean (sd)	13.49 (12.1)
Median [range]	10.0 [0–53]
Normal (n, %)	65 (92.9%)
Indication of PTSD (n, %)	5 (7.1%)
**Fear of cancer recurrence (FCRI-SF)**	
Mean (sd)	14.43 (8.2)
Median [range]	15.0 [0–34]
Normal	32 (45.7%)
Clinical fear of cancer recurrence—sensitivity (≥13)	38 (54.3%)
Clinical fear of cancer recurrence—specificity (≥16)	32 (45.7%)
Pathological fear of cancer recurrence (≥22)	15 (21.4%)
**Cognitive complaints (CFQ)**	
Mean (sd)	29.9 (15.4)
Median [range]	28.0 [0–83]
Normal (n, %)	57 (81.4%)
Elevated (n, %)	7 (10.0%)
Severe (n, %)	6 (8.6%)

## Data Availability

The datasets generated and analyzed during the study are available from the corresponding author upon reasonable request.

## Supplementary Materials

The following supporting information can be downloaded at: https://www.mdpi.com/article/10.3390/cancers16091638/s1, Figure S1: Consort diagram of the recruitment process.

## Permission to Reproduce from Other Sources

The graphical abstract has been designed using images from www.flaticon.com, accessed on 18 March 2024.
