# Supplementary figures and images for: Emotional Distress, Cognitive Complaints, and Care Needs among Advanced Cancer Survivors Treated with Immune Checkpoint Blockade: A Mixed-Method Study
Source: Cancers (Basel). 2024 Apr 24;16(9):1638. doi: 10.3390/cancers16091638 (PMC11083145; doi:10.3390/cancers16091638)

**Supplementary Figure S1. Consort diagram of the recruitment process**

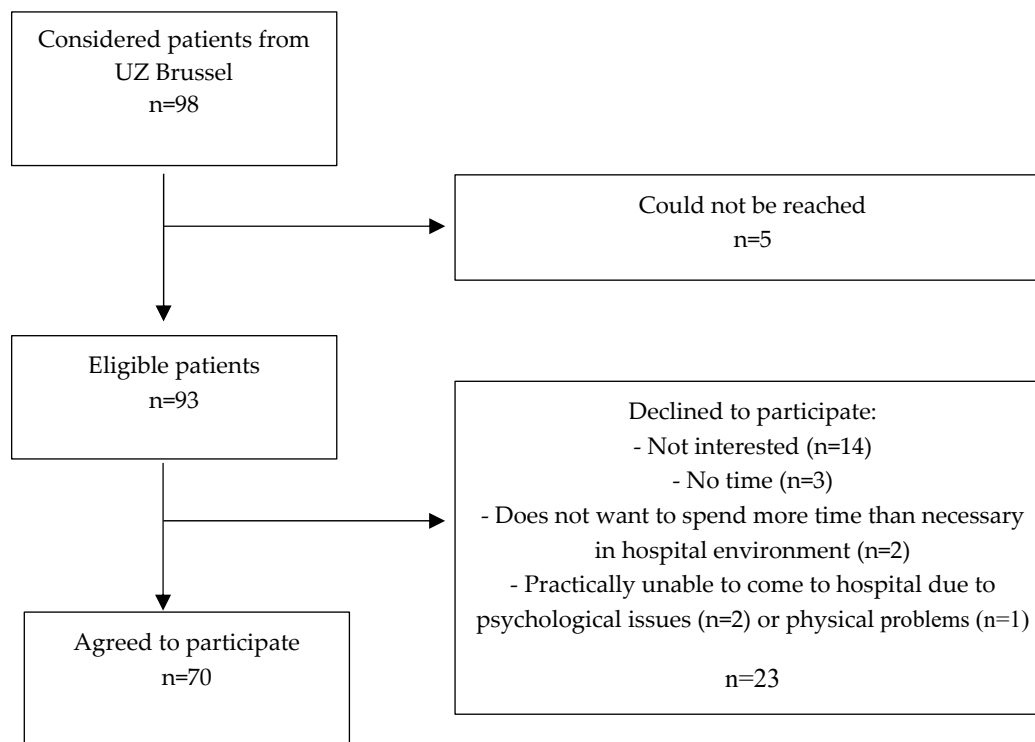

Supplement: Supplementary file 1 [file cancers-16-01638-s001.zip › cancers-2950170-supplementary.pdf]
